# Clonal Analysis in *Drosophila* Tissues With an Enhanced MAGIC Transgenesis Method

**DOI:** 10.21769/BioProtoc.5787

**Published:** 2026-08-20

**Authors:** Yifan Shen, Chun Han

**Affiliations:** 1 Department of Molecular Biology and Genetics, Cornell University, Ithaca, NY, USA; 2 Weill Institute for Cell and Molecular Biology, Cornell University, Ithaca, NY, USA

**Keywords:** *Drosophila melanogaster*, CRISPR/Cas9, Mosaic analysis, MAGIC, Mitotic recombination, Somatic clones, Genetic screens

## Abstract

Mosaic animals are highly valuable for investigating complex biological processes and cell lineages in vivo. Traditional mosaic techniques in *Drosophila*, such as the FRT/Flp system, rely on exogenous site-specific recombination sequences, preventing their application to unmodified mutant chromosomes or wild-derived strains. Mosaic analysis by gRNA-induced crossing-over (MAGIC) overcomes this limitation by utilizing the CRISPR/Cas9 system to generate targeted double-strand breaks (DSBs) that induce somatic homologous recombination in precursor cells. Here, we describe a comprehensive protocol for applying MAGIC with a newly developed, genome-wide MAGIC kit. This protocol utilizes optimized gRNA-markers with the Qtg2.1 scaffold for high-efficiency clone induction, alongside improved fluorescent labeling strategies for both positive MAGIC (pMAGIC) and negative MAGIC (nMAGIC). The procedure details the genetic crossing schemes, temporal induction of clones, and tissue processing for diverse *Drosophila* cell types. This method enables convenient mosaic analysis across all chromosomes and allows for the study of pericentromeric genes, deficiency chromosomes, and species-specific alleles in interspecific hybrids.

Key features

• **Recombinase-independent:** Generates somatic mosaic clones using CRISPR/Cas9 without requiring pre-inserted FRT sequences on the test chromosome.

• **Genome-wide application:** Includes a complete toolkit of pMAGIC and nMAGIC gRNA-markers optimized for all *Drosophila* chromosomal arms (X, 2L, 2R, 3L, 3R, and 4).

• **Optimized labeling:** Employs destabilized Gal80 for brighter pMAGIC clones and *tub-3xHA-BFP/IFP* for unambiguous visualization of nMAGIC clones.

• **Broad compatibility:** Applicable to a wide variety of tissues (e.g., neurons, glia, imaginal discs, polyploid tissues) and complex genetic backgrounds, including pericentromeric mutations and deficiencies.

## Graphical overview


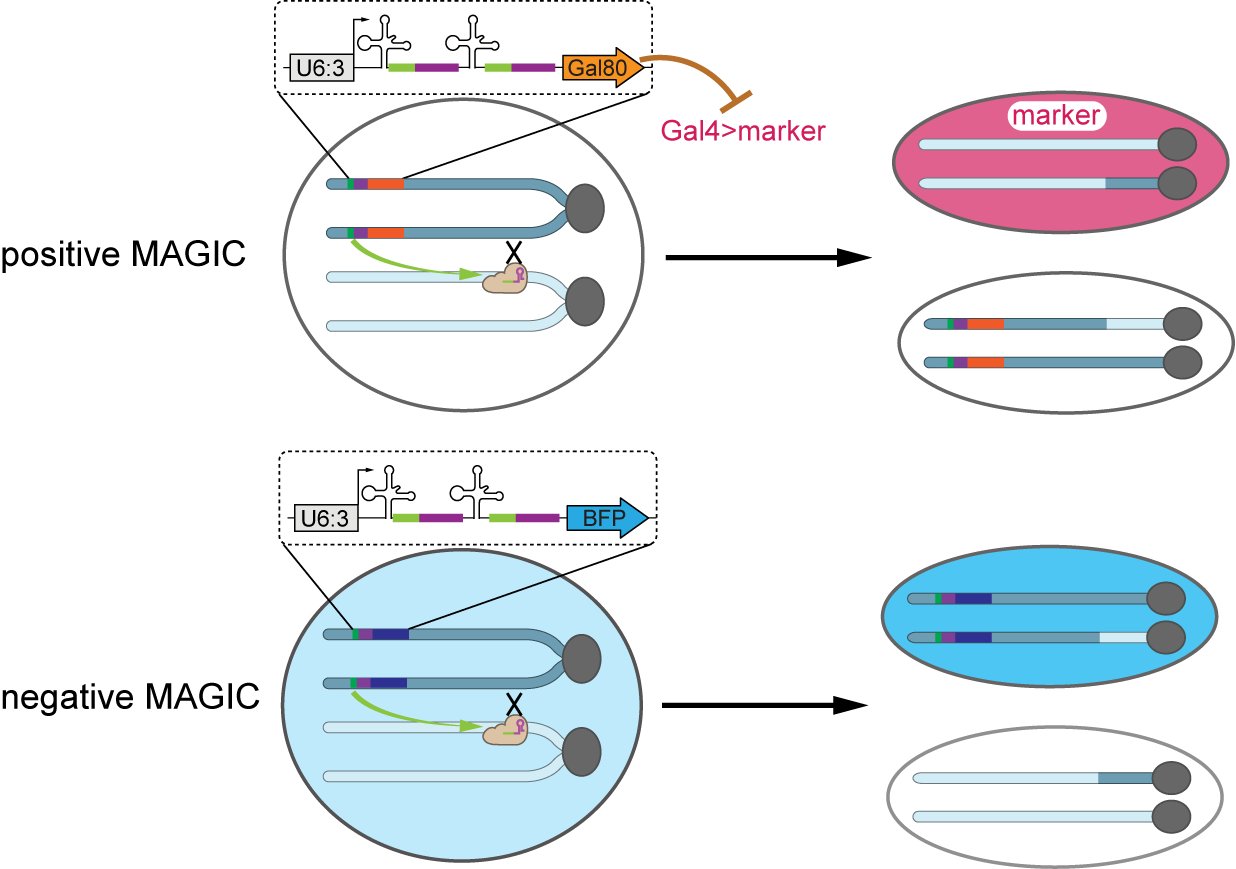


**Diagrams of positive MAGIC (pMAGIC) and negative MAGIC (nMAGIC).** The terms “positive” and “negative” define the visual labeling strategy used to identify the target mutant clones. pMAGIC (positive labeling) involves ubiquitously expressed Gal80 that suppresses Gal4-driven expression of a fluorescent marker in Gal80-containing cells. Only homozygous cells lacking the gRNA-marker will be labeled. nMAGIC (negative labeling) utilizes ubiquitously expressed blue fluorescent protein (BFP) to distinguish two populations of twin-spot cells, one with two copies of BFP and the other lacking BFP entirely. MAGIC, mosaic analysis by gRNA-induced crossing-over.

## Background

Mosaic analysis is a cornerstone of *Drosophila* genetics, classically driven by FRT/Flp site-specific recombination systems. However, these systems require the laborious introduction of recombination sites onto the specific chromosomal arms of interest. Consequently, existing mutant libraries, uncharacterized wild-derived strains, and genes located proximal to centromeres relative to established FRT sites remain largely inaccessible to standard clonal analysis.

To bypass these constraints, we recently established MAGIC (mosaic analysis by gRNA-induced crossing-over), which relies on CRISPR/Cas9 to generate double-strand breaks (DSBs) at predefined genomic locations, inducing homologous recombination during the S/G2 phase of dividing precursor cells. Segregation of these recombinant chromatids generates "twin spots," which can be visualized using carefully designed genetic markers.

While our initial proof-of-concept focused on chromosome 2L, this expanded protocol utilizes a comprehensive, genome-wide MAGIC kit. We have significantly optimized the gRNA-marker designs to overcome previous limitations of low clone frequency and weak fluorescence. The incorporation of the Qtg2.1 gRNA scaffold enhances DSB induction, while the use of a *tubulin* enhancer coupled with protein and mRNA destabilization sequences (DE) and an *SV40* polyA tail ensures complete suppression of Gal4 in heterozygous cells and bright labeling of pMAGIC clones. For nMAGIC, replacing nuclear blue fluorescent protein (BFP) with cytosolic 3xHA-BFP or infrared fluorescent protein (IFP) allows for clearer visualization of cell morphology. This protocol describes the genetic setups and subsequent imaging techniques to reliably generate and analyze mosaic clones across diverse *Drosophila* tissues.

## Materials and reagents

**Biological materials**

**Fly strains**

1. Cas9 driver lines: Tissue-specific, ubiquitous, or inducible Cas9 transgenic lines (Table 1)

**Table 1.** Cas9 driver lines

Cas9	Reference and source	Expression pattern in progenitor cells	Purpose
*vas-Cas9*	RRID: BDSC_55821	Germline and ubiquitous	To induce clones in any tissue
*zk-Cas9*	[1]; RRIDs: BDSC_607569 and BDSC_607570	Embryonic dorsal ectoderm	To induce clones in larval epidermis, imaginal discs, larval sensory neurons, and motor neurons
*ey-Cas9*	[2]; RRID: BDSC_606039	Eye and antennal discs	To induce clones in cells derived from the eye and antennal discs
*hs-Cas9*	[3]	Ubiquitous (inducible)	To induce clones in any tissue
*gcm-Cas9*	[4]; RRID: BDSC_606774	Glial progenitor	To induce clones in glia
*Act5C-Cas9*	[5]; RRID: BDSC_54590	Ubiquitous	To induce clones in any tissue
*tub-Cas9*	[6]; RRID: BDSC_81930	Ubiquitous	To induce clones in any tissue
*hh-Cas9*	[7]; RRIDs: BDSC_81927, BDSC_81928, and BDSC_81929	Embryonic epidermal cells; imaginal tissues	To induce clones in imaginal discs

2. MAGIC gRNA-marker lines: Positive MAGIC (pMAGIC) or negative MAGIC (nMAGIC) lines from a genome-wide kit covering all chromosomal arms (X, 2L, 2R, 3L, 3R, and 4)

*Note: The complete list of Bloomington* Drosophila *Stock Center (BDSC) catalog numbers and corresponding target sequences for all lines are provided in Table S1.*

3. Fluorescent reporter lines (required for pMAGIC, optional for nMAGIC): Standard Gal4/UAS reporter lines (e.g., tub-Gal4, UAS-mCD8-GFP, UAS-tdTom)

4. User-defined mutant lines: Any *Drosophila* line carrying a mutation of interest or a chromosomal deletion

**Critical:** The mutation or deleted segment must be located on the same chromosomal arm and distal to the chosen MAGIC gRNA target site.

## Materials and reagents

1. Flies can be cultured on any commonly used *Drosophila* medium, such as the Bloomington formulation yeast–cornmeal–molasses medium (e.g., Nutri-Fly, Genesee Scientific, catalog number: 66-112) and commercial ready-made medium (such as LabExpress fly food B)

2. Genetic crosses can be set up in standard fly culture vials (e.g., Genesee Scientific, catalog number: 32-113)

3. 60 mm Petri dishes (VWR, catalog number: 25373-085)

4. PYREX^®^ 9-depression glass spot plate (Corning, catalog number: 7220-85)

## Equipment

1. Standard stereomicroscope for sorting genetic markers and balancing flies

2. Fluorescence-equipped stereomicroscope (e.g., Nikon, model: SMZ18) for in vivo prescreening and direct visualization of fluorescent clones in live animals

3. Incubators (capable of maintaining 18 and 25 °C) with a 12/12 light/dark cycle; 18 °C is used for preventing activation of hs-Cas9, while 25 °C is for the standard culture of *Drosophila*

4. Water bath (e.g., VWR, catalog number: 10128-126) set to 37 °C for activating *hs-Cas9*

5. Fly pushing pads (e.g., Genesee Scientific, catalog number: 59-172) connected to CO_2_ delivery systems and fine-tipped paintbrushes (e.g., Thermo Fisher Scientific, catalog number: 03-665A) for fly sorting

## Procedure

**A. Experimental design and component selection**

*Note: The success of MAGIC relies on correctly pairing four genetic components: Cas9, gRNA-marker, fluorescent reporters, and the test mutation. This system does NOT require FRT sites on the test chromosome.*

1. Define the crossover site: Determine the chromosomal location of your mutant gene. Select a MAGIC gRNA-marker line targeting a pericentromeric region proximal to your gene.

*Note: The genome-wide kit provides three target sites per arm with varying efficiencies. Select a high-efficiency gRNA for dense clone generation or a low-efficiency gRNA for sparse clones.*

2. Select the Cas9 driver: Choose a Cas9 line expressed in the precursor cells of your target tissue (Table 1).

a. Use ubiquitous Cas9 (*vas-Cas9, Act5C-Cas9*) for broad multi-tissue clones.

b. Use enhancer-driven Cas9 (e.g., *ey-Cas9, zk-Cas9*) for tissue-specific induction.

c. Use *hs-Cas9* for precise temporal control of mitotic recombination.

3. Choose the MAGIC modality (pMAGIC vs. nMAGIC):

a. pMAGIC (positive labeling) uses a Gal80 marker [e.g., *tub-Gal80(DE)*] to repress Gal4 in heterozygous cells. Clones homozygous for the mutant arm lose Gal80 and become positively labeled by the Gal4-driven fluorescent reporter.

b. nMAGIC (negative labeling) uses a ubiquitously expressed BFP or IFP (e.g., *tub-3xHA-BFP*). Clones homozygous for the mutant arm are identified by the absence of the fluorescent marker (loss of BFP/IFP).

**B. Genetic crossing schemes**

**Caution:** Do not maintain Cas9 and gRNA transgenes together in the same stock long-term, as leaky germline Cas9 activity can prematurely mutate the gRNA target site.

**Scheme 1: Generating pMAGIC clones**

1. P0 cross (building the components): Place 10–15 virgin females carrying the gRNA-marker and reporter components into a vial alongside 5–8 males carrying the Cas9 and the user-defined mutant allele. Allow the flies to mate and reproduce at 25 °C. Transfer the flies to a new vial after three days initially and then every day to prevent crowding of progeny in the vials.

a. Female genotype example: *gRNA-marker(Gal80)/Balancer 1; Gal4, UAS reporter/Balancer 2*

b. Male genotype example: *mutant_allele/Balancer 1; Cas9/Balancer 2*

2. F1 generation (clone induction): Maintain the P0 cross at 25 °C. The somatic crossing-over events will occur during the development of the F1 progeny.

a. Target F1 genotype: *gRNA-marker(Gal80)/mutant_allele; Gal4, UAS reporter/Cas9*

b. Mechanism: In the precursor cells of the F1 animal, Cas9 induces a DSB at the gRNA target site. Following G2-X segregation, the resulting homozygous mutant cell (*mutant_allele/mutant_allele*) will lack the gRNA-marker (Gal80) transgene. Without Gal80, the Gal4 component activates the UAS reporter, permanently illuminating the mutant clone.

**Scheme 2: Generating nMAGIC clones**

1. P0 cross: Following the same physical setup parameters as in Scheme 1, place 10–15 virgin females carrying the gRNA-marker into a vial with 5–8 males carrying the Cas9 and the mutant allele. Transfer the flies to new vials as needed.

a. Female genotype example: *gRNA-marker(BFP)/gRNA-marker(BFP)*

b. Male genotype example: *mutant_allele/Balancer 1; Cas9/Balancer 2*

2. F1 generation (clone induction):

a. Target F1 genotype: *gRNA-marker(BFP)/mutant_allele; Cas9/+*

b. Mechanism: Homozygous mutant clones generated via somatic recombination will lack the gRNA-marker (BFP) transgene and will be visualized as distinct "dark" (BFP-negative) patches amidst the intermediate (heterozygous) and bright (homozygous wild-type twin spot) background.

**Figure 1.**
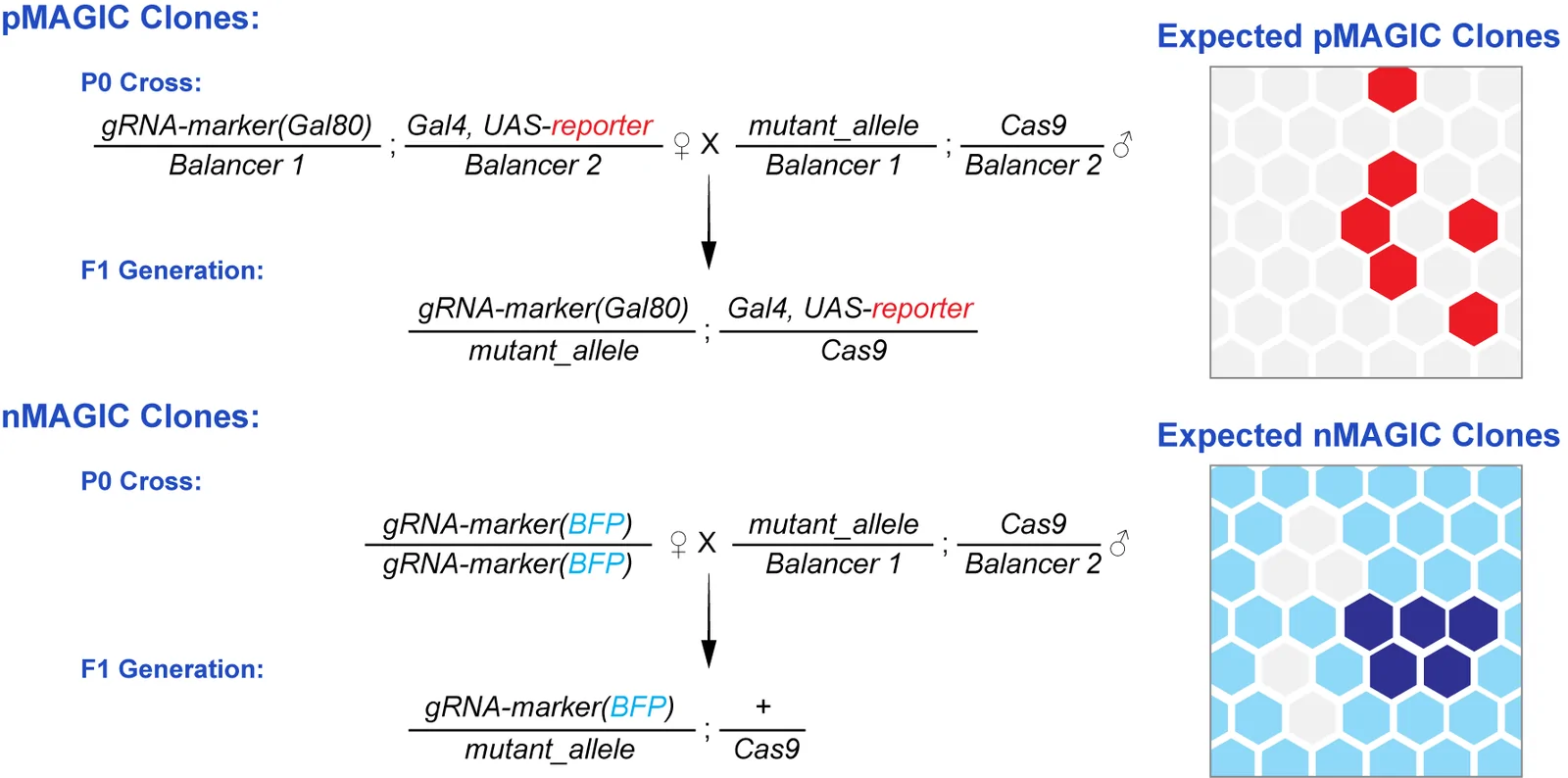
Genetic crossing schemes and expected visual readouts for pMAGIC and nMAGIC. (Top) Positive MAGIC (pMAGIC). The schematic details the P0 cross and the resulting F1 genotype. In F1 somatic cells, Cas9-induced homologous recombination generates homozygous mutant cells that lose the gRNA-marker (Gal80) transgene. As illustrated in the right panel, the de-repression of Gal4 activates the UAS reporter, allowing the mutant clones to be "positively" labeled (e.g., brightly fluorescent red cells) against a non-fluorescent heterozygous background. (Bottom) Negative MAGIC (nMAGIC). The lower panel outlines the crossing strategy to generate nMAGIC clones. Following somatic recombination in the F1 progeny, the resulting homozygous mutant clones lack the gRNA-marker (BFP) entirely. The schematic on the right demonstrates this "negative" labeling readout: mutant clones appear as distinctly dark (BFP-negative) cell patches, which are easily distinguishable from the intermediate (heterozygous, light blue) background and the bright (homozygous wild-type twin spot, white) cells. MAGIC, mosaic analysis by gRNA-induced crossing-over.

Genetic crossing schemes and expected visual readouts for MAGIC clone induction are shown in Figure 1.

**C. Temporal clone induction via heat shock (optional)**

*Note: Temporal control of clone induction is achieved by activating hs-Cas9 via heat shock. Consult Table 2 to determine the timing of the heat shock based on the specific developmental stage at which you wish to activate Cas9. Since hs-Cas9 has leaky activity at room temperature, for tight temporal control, the target genotype should be kept at 18 °C until the desired developmental stage has been reached.*

**Table 2.** Heat shock timing at different developmental stages

Developmental stage	Approximate time at 18 °C (hours after egg laying, AEL)
Embryogenesis ends/first instar begins	48 h
Second instar larva	96 h
Third instar larva	144 h
Pupariation	240 h

1. Allow the F1 animals from the P0 cross to develop at 18 °C until they reach the desired developmental stage (e.g., first instar larvae).

2. Submerge the vials in a 37 °C water bath for 1 h. Extend this duration if too few clones are induced; shorten this duration if too many animals die after the heat shock.

3. Immediately return the vials to an 18 °C incubator and allow the F1 animals to continue development.

**D. Animal screening and downstream assay**

1. To prepare F1 larvae for screening, gently pick them up from the culture medium using a pair of forceps. Rinse the larvae with water in a 60 mm Petri dish to remove food debris. Transfer the cleaned larvae into a water-filled well on a 9-depression glass spot plate. Screen for larvae that do not contain any balancer chromosome under a standard or fluorescence stereomicroscope. Transfer the larvae of the correct genotype to another well for downstream assays.

2. To screen F1 pupae, examine them under a standard or fluorescence stereomicroscope while they are attached to the wall of the culture vial. Mark those that do not contain any balancer chromosome. Gently pick them up using a pair of forceps or a moistened brush for downstream assays.

3. To screen F1 adults, knock them out on a CO_2_ flypad. Examine them under a standard or fluorescence stereomicroscope. Pick those that do not contain any balancer chromosome for downstream assays.

4. Proceed with your desired downstream phenotypic assay on these mosaic animals (e.g., confocal imaging of intact larvae or dissected tissues).

## Data analysis

1. Clone frequency assessment: To evaluate the efficiency of a specific Cas9/gRNA-marker combination, count the number of independent fluorescent (pMAGIC) or non-fluorescent (nMAGIC) clones within a defined anatomical region (e.g., segments A1–A7 in larvae) using a fluorescence stereomicroscope.

2. Phenotypic comparison: Compare the phenotype (e.g., viability, developmental timing, structural morphology) of mosaic animals carrying homozygous mutant clones against control mosaic animals generated using wild-type chromosomes.

3. Statistical analysis: Analyze clone counts or phenotypic penetrance using standard statistical software (e.g., RStudio). Apply Student's t-tests for pairwise comparisons or one-way ANOVA for multiple gRNA efficiency comparisons.

## Validation of protocol

This protocol (or parts of it) has been used and validated in the following research articles:

• Shen et al. [8]. A genome-wide MAGIC kit for recombinase-independent mosaic analysis in *Drosophila. eLife* (2026). DOI: 10.7554/eLife.108453. To view the functional validation strategy—including quantitative comparisons of clone induction frequencies across different chromosomal arms and tissue types (such as wing discs and larval brain)—refer specifically to Figures 2, 3, and Supplementary Figure S1 in this publication.

• Allen et al. [1]. Versatile CRISPR/Cas9-mediated mosaic analysis by gRNA-induced crossing-over for unmodified genomes. *PLOS Biology* 19(1): e3001061 (2021). DOI: 10.1371/journal.pbio.3001061. Validation of the core MAGIC principle, mapping its efficiency in sensory dendrite arborization (da) neurons, was documented in Figures 1–4 of this study.

## General notes and troubleshooting

**General notes**

1. Transgene variegation on chromosome 4: Transgenes inserted on the fourth chromosome (such as the nMAGIC *tub-3xHA-BFP* marker) may exhibit uneven or variegated expression in certain tissues due to heterochromatin silencing. pMAGIC is generally recommended for neuronal analysis on the fourth chromosome as the Gal80 repressor remains highly efficient.

2. Off-target considerations: Because perfect DSB repair recreates the gRNA target site, Cas9 will repeatedly cut until an indel forms. Even cells that do not undergo mitotic recombination will likely carry an indel at the gRNA locus. Therefore, the gRNA target sites in this kit were specifically selected within non-critical intergenic sequences to prevent confounding phenotypic artifacts.

**Troubleshooting**

**Problem 1:** The desired genotype is difficult to obtain in the F1 generation.

Possible cause: Too many homozygous clones of a lethal mutation in critical tissues could kill animals at an early developmental stage.

Solution: Switch to a tissue-specific Cas9 driver to restrict DSB induction solely to the somatic tissue of interest.

**Problem 2:** No clones observed in the F1 generation.

Possible cause 1: The chosen gRNA target sequence has inherently low cleavage efficiency.

Solution: Switch to a different MAGIC gRNA-marker line targeting a different locus on the same chromosomal arm (e.g., switch from *gRNA-40E1* to *gRNA-40D2*).

Possible cause 2: The Cas9 driver is not expressed in the appropriate precursor cells during the G2 phase.

Solution: Validate the temporal and spatial expression pattern of your chosen Cas9 driver, or switch to an inducible *hs-Cas9* and optimize the timing of the heat shock.

**Problem 3:** Difficulty in identifying larvae of the correct genotype-carrying clones.

Possible cause: Identifying clone-carrying larvae can be difficult if the clones are not brightly labeled. Additionally, unlike traditional FRT-based mosaic methods, mitotic recombination can be induced between a gRNA-marker chromosome and a balancer chromosome in the MAGIC system. Therefore, simply observing a clone in a larva does not guarantee it has the correct experimental genotype.

Solution: When screening at the larval stage, it is critical to use balancer chromosomes equipped with markers that can be easily distinguished in larvae (such as Tubby or specific fluorescent reporters). This allows for accurate negative selection; without these larval-specific balancers, there is no reliable way to distinguish the desired experimental larvae from those carrying the balancer chromosome.

## Supplementary information

The following supporting information can be downloaded here:

1. Table S1. Comprehensive list of genome-wide MAGIC gRNA-marker lines, target sites, and BDSC catalog numbers.
